# Transient blindness associated with mild encephalitis/encephalopathy with a reversible splenial lesion (MERS): a case report and review of literature

**DOI:** 10.1186/s13052-020-00918-0

**Published:** 2020-10-12

**Authors:** Antonella Tuscano, Marisa Zoppo, Carlotta Canavese, Maurizio Cogoni, Carlo Scolfaro

**Affiliations:** 1 Department of Pediatric and Public Health Sciences, Postgraduate School of Pediatrics, Regina Margherita Children’s Hospital, University of Turin, Turin, Italy; 2 Department of Pediatrics, Infectious Diseases Unit, University of Turin, Regina Margherita Children’s Hospital, Turin, Italy; 3 Division of Child and Adolescent Neuropsychiatry, Department of Public Health and Pediatric Sciences, University of Turin, Turin, Italy; 4 Department of Neuroradiology, CTO Hospital, Torino, Italy; 5 No more needed, Turin, Italy; 6 No more needed, Italia

**Keywords:** Reversible encephalopathy, Transient blindness, Corpus callosum

## Abstract

**Background:**

Mild encephalitis/encephalopathy with a reversible splenial lesion (MERS) is a clinical-radiological syndrome that can be related to infectious and non-infectious conditions. The most prominent neurological symptoms are disturbance of consciousness, abnormal speech, delirious behavior, seizures, muscle weakness, ophthalmoplegia, facial nerve paralysis and headache. Here we report the case of a child with MERS presenting with the unusual symptom of bilateral transient blindness.

**Case presentation:**

A 4-year-old female patient, with a history of fever, abdominal pain, loss of appetite and cough lasted for a few days, experienced 3 episodes of transient bilateral loss of vision with difficulty in walking. Her physical examination showed absence of focal neurological and meningeal irritation signs, although responsiveness was slightly impaired. The ophthalmologic evaluation, including a fundus oculi examination, was negative.

The electroencephalogram showed slow activity in the temporo-occipital regions, more evident in the right hemisphere. A lumbar puncture was performed and cerebrospinal fluid analysis revealed normal glycorrhachia, cell counts, protein levels and IgG index.

Magnetic resonance imaging of the brain showed a signal alteration in the splenium of the corpus callosum, without contrast enhancement. This finding was suggestive of a reversible cytotoxic lesion. Empiric antiviral treatment with acyclovir and intravenous dexamethasone was initiated. Polymerase chain reaction search for neurotropic viral nucleic acid sequences in the cerebrospinal fluid was negative, while a low number of HHV-6 DNA copies was detected in the blood.

Electroencephalograms were repeated in the following days, showing a progressive normalization of the pattern. The child was discharged without symptoms after 10 days of treatment with oral corticosteroids. After 40 days, brain magnetic resonance imaging showed a complete normalization of the signal alteration in the splenium of the corpus callosum.

**Conclusion:**

Transient blindness was reported as an initial symptom of MERS in a few children. To date, there is no evidence of effective treatment methods. Nonetheless, MERS diagnosis provides pediatricians with valuable prognostic information in order to reassure patients and their families about the good outcome of this disease.

## Background

Mild encephalitis/encephalopathy with a reversible splenial lesion syndrome (MERS) is a clinical-radiological syndrome mostly reported in East Asian populations. It generally correlates with good prognosis and its typical neurologic symptoms are delirious behavior, decreasing conscious level and seizures, always followed by prodromal illness such as fever, cough, vomiting or diarrhea. The pathogenesis is unknown, and infections remain the most common cause. During acute MERS episodes, MRI generally reveals a lesion in the splenium of the corpus callosum (SCC), sometimes also extending to other areas of the corpus callosum or adjacent parenchyma [1]. Diseases and conditions characterized by a reversible isolated lesion in the SCC with a transient and abnormal diffusion restriction detected by MRI are commonly called “reversible splenial lesion syndromes” (RESLES), independently of their etiologies. Despite its inclusion in the wide range of RESLES, MERS is mostly reported in children and is characterized by a splenial lesion usually attributed to an infection and, at clinical presentation, by a mild encephalitis/encephalopathy [2].

Here we describe a case of MERS in an Italian 4-year-old female patient, with a cytotoxic lesion in the SCC detected by MRI and the unusual clinical presentation of acute transient bilateral blindness.

## Case presentation

A 4-year-old female patient presented with a history of fever, abdominal pain, loss of appetite and cough for a few days. She was examined by her pediatrician and oral amoxicillin-clavulanate was prescribed due to suspected bronchitis. The morning after the examination, she experienced 3 episodes of bilateral loss of vision with difficulty in walking. The symptoms were transient and lasted a few seconds. Therefore, she was admitted to the hospital emergency department.

Her family history was unremarkable, with the exception of a grandmother affected by syringomyelia and Chiari malformation.

Before her hospitalization, the child had always been in good general health, except for frequent episodes of otitis, with the last episode occurring in the previous month. After the last episode, a prophylaxis for otitis with azithromycin was prescribed by her pediatrician.

On physical examination, the patient was in good general condition. The girl was collaborative during the medical evaluation, although her responsiveness was slightly impaired. Neither focal neurological signs nor meningeal irritation signs were observed.

Laboratory tests showed mild hypoglycemia (glucose serum level = 59 mg/dl) and mild hyponatremia (134 mmol/L). An ultrasound of the abdomen was performed because of the abdominal pain. No pathological signs were found. The episodes of amaurosis prompted an ophthalmologic evaluation, including a fundus oculi examination that was negative.

On the following day, a maculopapular rash appeared on the patient’s face and chest. Nevertheless, it was transient and quickly disappeared without specific treatment.

The electroencephalogram (EEG) showed slow activity on the temporo-occipital regions, more evident in the right hemisphere. The child neurologist observed a mild generalized hypotonia without significant neurological deficits.

The child was then admitted to perform a brain and spine MRI and a lumbar puncture, in order to exclude the presence of viral encephalitis, cerebral abscesses or other cerebral inflammatory lesions, such as acute disseminated encephalomyelitis (ADEM).

**Fig. 1:**
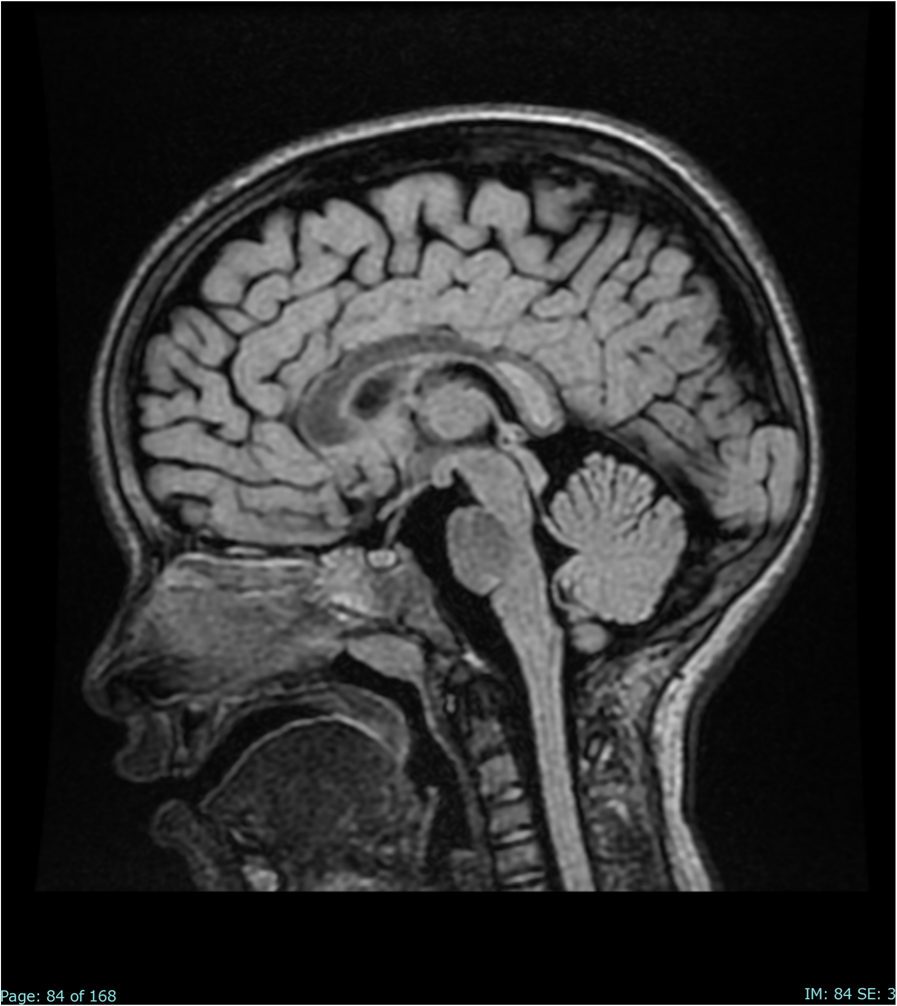
Sagittal FLAIR image on the day of admission shows a focal high-signal lesion in the splenium of the corpus callosum

MRI with diffusion-weighted imaging (DWI) of the brain and spine showed a signal alteration in the SCC, without contrast enhancement and with low apparent diffusion coefficient (ADC) values, thus suggesting an abnormal diffusion restriction and a reversible cytotoxic lesion (Fig. 1).

Antiviral treatment with intravenous acyclovir (10 mg/Kg every 8 h) and dexamethasone (0.2 mg/Kg every 8 h) was initiated, while waiting for the results of the polymerase chain reaction (PCR) search for neurotropic viruses, bacteria and fungi in the cerebral spinal fluid (CSF) and peripheral blood (*Streptococcus pneumoniae*, Neisseria meningitidis, *Haemophilus influenzae*, *Streptococcus agalactiae*, *Listeria monocytogenes*, *Escherichia coli* K1, cytomegalovirus, herpes simplex 1, herpes simplex 2, human herpesvirus-6 (HHV-6), enterovirus, human parechovirus, varicella-zoster, *Cryptococcus neoformans*, and adenovirus). Antiviral therapy was suspended because the polymerase chain reaction search for neurotropic viral nucleic acid sequences in the cerebrospinal fluid and peripheral blood was negative. A nasal swab for influenza virus was also negative. A low number of HHV-6 DNA copies was detected in the blood, but this data did not change patient’s management.

Collateral findings detected by MRI were pansinusitis and bilateral otomastoiditis, but no specific otolaryngologic treatment was considered.

EEGs were repeated in the following days, showing a progressive normalization of the pattern.

The child has always been in good general condition and apyretic. As a consequence, she was discharged after 10 days of admission and treatment with oral corticosteroids, with a progressive reduction of the steroid dose.

**Fig. 2:**
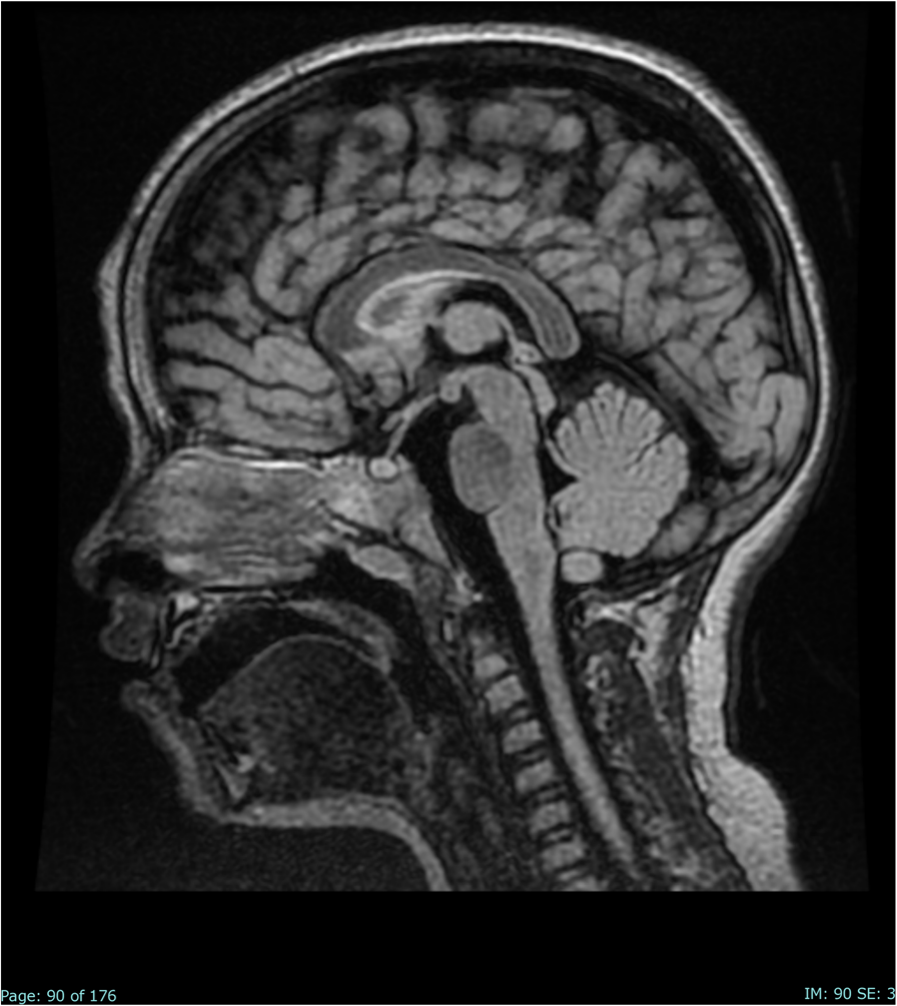
Sagittal FLAIR image on day 40 after admission: the lesion in the splenium of the corpus callosum has completely disappeared

After 40 days, a follow-up brain MRI was repeated, showing a complete normalization of the signal alteration in the SCC (Fig. 2). The EEG was also repeated, showing a complete normalization of the pattern.

After 2 years of follow-up, the child was in good general condition, without neurological deficits.

## Review of the literature

MERS is a rare clinical-radiological syndrome with unknown pathogenesis, mostly reported in the East Asian populations. This syndrome was first described by Tada et al. in 2004 [2]. It usually develops in children between the ages of 0 and 16 years and only occasionally in adults. Infections are considered the main trigger of the disease.

### Genetic predisposition

Almost all cases of MERS have been reported in East Asian populations (mostly in Japan), thus suggesting a genetic predisposition in this population [3]. To date, the genetic factors involved in the development of MERS are unknown. Gatto et al. recently described two cases of MERS, associated with echovirus 6 and influenza A infection, in two twin sisters at the age of 4 years. Exome sequencing was performed on samples from the twins to potentially identify the causative gene and 2 different frameshift mutations of the CD36 gene were found in both twins [4].

### Pathogenesis

Infections are the most common etiologies of reversible splenial lesions in children.

The major pathogens associated with MERS are viruses, such as influenza virus (A and B), mumps virus, Epstein–Barr virus, herpes simplex virus, parvovirus B-19, cytomegalovirus, dengue virus, adenovirus, and rotavirus. In a study reported by Fang et al., 16/29 (55%) MERS pediatric patients had viral diseases, and rotavirus was the most common pathogen [1]. Bacteria should also be considered, such as Streptococci, Mycoplasma pneumoniae, *Escherichia coli*, Klebsiella pneumonia, and *Enterococcus faecalis* [5, 6]. Recently, a case of MERS in a 55-year-old Japanese man with cerebral malaria was reported as well [7].

Moreover, cases of MERS were observed in patients with Kawasaki disease [8]. Oger et al. reported a pediatric case of MERS associated with autoimmune glial fibrillary acidic protein (GFAP) astrocytopathy, a rare antibody-mediated encephalitis [9].

Non-infectious conditions related to reversible splenial lesions are seizures, antiepileptic drug withdrawal, metabolic disturbances, and renal or hepatic dysfunction [10].

To date, a common pathophysiological mechanism explaining selective splenial involvement has not been found. However, there are several hypotheses on MERS pathogenesis, including intramyelinic edema, hyponatremia, axonal damage, and oxidative stress [11].

Many MERS patients without any attributed etiologic agent in CSF cultures were reported to have other foci of infection. Recently, several cases of MERS with accompanying acute focal bacterial nephritis (AFBN) were reported [12]. In this regard, a further explanation for transient cerebral edema is based on the presence of myelin-specific neurotoxin released by a pathogen causing inflammatory infiltrates [13].

### Clinical presentation

MERS is typically characterized by a prodromal illness consisting of fever, cough and digestive tract symptoms such as vomiting, diarrhea or abdominal pain, followed 1–7 days later by encephalopathy [3, 14]. The neurological features of MERS include disturbance of consciousness, abnormal speech, delirious behavior, headache, agitation, disorientation, seizures, facial nerve paralysis, and nuchal rigidity. Usually, all of these symptoms completely recover within a month after presentation. The most common neurological symptom described in the literature is delirious behavior with altered consciousness, which may present as akinetic mutism [10, 13].

In a retrospective study and summary of case series, Chen et al. analyzed the clinical features of 15 children affected by MERS and found that the clinical symptoms associated with a more severe presentation were altered consciousness (drowsiness, stupor) and motor deterioration [11].

In our case, the predominant neurological symptom was bilateral transient blindness, which in the literature was reported as an initial symptom of MERS in a few children (Table 1).

**Table 1:** Main neurological symptoms reported at clinical presentation in MERS cases

Study	Number of cases	Neurological symptoms, N (% of total cases)										
		Consciousness disturbance (irritability, lethargy, drowsiness, delirium)	Seizure	Headache	Dysphagia, dyslalia, slurred speech	Acute urinary retention	Ataxia or motor disorders	Neck stiffness	Hallucinations	Dizziness	Blurred vision	Blindness
Fang Q. et al. [1]	29	18/29 (62%)	18/29 (62%)	4/29 (14%)	1/29 (3%)	1/29 (3%)	1/29 (3%)	0/29 (0%)	0/29 (0%)	0/29 (0%)	0/29 (0%)	**0/7 (0%)**
Pan J.J. et al. [14]	27	11/27 (41%)	5/27 (19%)	14/27 (52%)	1/27 (4%)	2/27 (7%)	1/27 (4%)	0/27 (0%)	0/27 (0%)	1/27 (4%)	2/27 (7%)	**0/27 (0%)**
Tada H. et al. [2]	15	12/15 (80%)	8/15 (53%)	2/15 (13%)	0/15 (0%)	0/15 (0%)	2/15 (13%)	1/15 (7%)	1/15 (7%)	2/15 (13%)	0/15 (0%)	**1/15 (7%)**
Ka A. et al. [3]	7	7/7 (100%)	1/7 (14%)	0/7 (0%)	3/7 (43%)	0/7 (0%)	4/7 (57%)	0/7 (0%)	2/7 (29%)	0/7 (0%)	0/7 (0%)	**0/7 (0%)**
Chen W.-X. et al. [11]	15	6/15 (40%)	12/15 (80%)	2/15 (13%)	2/15 (13%)	0/15 (0%)	5/15 (33%)	1/15 (7%)	1/15 (7%)	1/15 (7%)	0/15 (0%)	**0/15 (0%)**
Yildiz A.E. et al. [13]	8	2/8 (25%)	4/8 (50%)	1/8 (12,5%)	2/8 (25%)	0/8 (0%)	1/8 (12,5%)	2/8 (25%)	0/8 (0%)	0/8 (0%)	0/8 (0%)	**2/8 (25%)**
Winslow H. et al. [15]	1	0/1 (0%)	0/1 (0%)	0/1 (0%)	0/1 (0%)	0/1 (0%)	0/1 (0%)	0/1 (0%)	1/1 (100%)	0/1 (0%)	0/1 (0%)	0/1 (0%)
Lu P.L. et al. [16]	15	3/15 (20%)	3/15 (20%)	3/15 (20%)	1/15 (7%)	0/15 (0%)	1/15 (7%)	0/15 (0%)	0/15 (0%)	3/15 (20%)	2/15 (13%)	0/15 (0%)

*Abbreviations*: *MERS* Mild encephalitis/encephalopathy with a reversible splenial lesion, *N* Number

The disturbance in the connections of the corpus callosum may cause disorders of motor control, spatial orientation, vision, hearing, and language-related behaviors [17]. These disorders may explain the neurological symptoms of MERS, including blindness [13].

### Neuroradiological imaging

On MRI, MERS is almost always associated with a transient splenial lesion that is slightly hyperintense on T2-weighted images and isointense to slightly hypointense on T1-weighted images, and that shows reduced diffusion without contrast enhancement during the acute period of the disease. Splenial lesions may extend into the callosal radiations, the frontoparietal subcortical white matter, the rest of the corpus callosum, and even the cerebellum. A classification of MERS based on MRI data was proposed [18, 19]. In MERS type 1, the lesions are limited to the splenium (ovoid or band shaped), as observed in our case report, whereas in MERS type 2 the lesions are not limited to the splenium. Clinical and radiological outcome is usually favorable with clinical improvement occurring within 1–2 days, while radiologic improvement within 10 days-4 months [13]. Rarely, patients, especially those with type-2 lesions on MRI, may develop neurological sequelae. Lesions may persist on MRI for months even if their size diminishes [13].

### Laboratory findings

Usually, raised serum inflammatory markers (white cell count and C-reactive protein) in the absence of CSF inflammation can be found in children diagnosed with MERS, thus supporting the hypothesis that this syndrome is an infection-associated encephalopathy rather than an encephalitis [3]. Morichi et al. investigated the cytokine profiles in CSF and serum from a child with MERS during influenza infection, and compared them with those of another serious type of influenza-associated encephalopathy. There was no elevation of Interleukin (IL)-1 β. In the early phase, the inhibitory cytokines of IL-10 and IFN-γ were elevated in the CSF. The elevations of the cytokine levels were generally mild, as compared with those in other patients [20].

Serum sodium levels were significantly lower in patients with MERS than in age-matched controls. These data support the hypothesis that a transient cerebral edema may develop as a result of hyponatremia [3]. The EEG may be abnormal in the acute phases, but returns to normal with clinical recovery. In a literature review, Pan et al. observed that 10 patients out of 27 had an EEG with global diffuse slow waves during recording [14].

The differential diagnosis of MERS includes infections, ischemia, multiple sclerosis, lymphoma, ADEM, and posterior reversible encephalopathy.

### Therapeutic options

MERS is a rare disease and no high-level evidence on the therapeutic approaches is currently available. Methylprednisolone pulse therapy and intravenous immune globulin (IVIG) are recommended for patients with infectious encephalopathy, regardless of pathogen or clinical-radiological syndromes. No evidence of efficacy of these treatments on MERS is available. Pan et al. reported that only 3 patients out of 27 were treated with methylprednisolone pulse therapy and IVIG. However, all 27 MERS patients achieved a complete clinical recovery, regardless of treatment administration. This suggests that methylprednisolone pulse therapy or IVIG may not always be necessary [14].

## Discussion and conclusions

In our case, the unusual main neurological symptom was acute transient bilateral blindness, which appeared after a few days with symptoms suggestive of viral infection (fever, abdominal pain, cough, skin rash). The slight HHV-6 positivity detected by PCR in the blood remains a result of uncertain interpretation within the clinical picture of this patient.

In this case, there are several reasons why HHV-6 was not strongly considered in the etiology. Sometimes, a slight HHV-6 positivity by PCR may also be found in patients without related symptoms. The 4-year-old child presented with non-specific symptoms, which could be attributed to a variety of viruses, and HHV-6 disease usually occurs within the first 2 years of life. Furthermore, in this case, the PCR search for HHV-6 in the CSF was negative.

EEG abnormalities were not global diffuse slow waves, but focal slow waves in the temporo-occipital regions, especially in the right hemisphere.

MRI imaging showed lesions limited to the splenium (MERS type 1), and MRI repeated 40 days later showed that these lesions had resolved. Pansinusitis was an additional neuroradiological finding and its etiologic link with MERS in this particular case remains to be clarified.

With regard to laboratory findings, our patient had mild hyponatremia at hospital admission (134 mmol/L). However, this result only provides a limited contribution to the clinical presentation.

Our patient was treated with intravenous antiviral therapy until the PCR search for neurotropic viral nucleic acid sequences in the blood and CSF was found to be negative. In addition, the patient received corticosteroid therapy, which was continued after discharge with a progressive dose reduction. To date, there is no evidence of an effective treatment for patients with MERS, and the prognosis, especially of MERS type 1, seems to be favorable regardless of the type of therapy.

In children, MERS shows a wide spectrum of clinical presentations involved. Visual disturbances are rare symptoms of this syndrome, which in most cases presents with impaired consciousness (drowsiness, stupor) and motor deterioration. Most of the MERS cases show a favorable outcome regardless of treatment. The early recognition of this condition in children with encephalopathy may limit unnecessary and potentially toxic treatments. Moreover, although the disease presentation may be severe and worrisome, MERS diagnosis allows pediatricians to reassure patients’ families about the good outcome of this disease.

## Data Availability

Data sharing not applicable to this article as no datasets were generated or analyzed during the current study.

## Abbreviations

ADEM: Acute disseminated encephalomyelitis
ADC: Apparent diffusion coefficient
AFBN: Acute focal bacterial nephritis
CSF: Cerebral spinal fluid
DWI: Diffusion-weighted imaging
EEG: Electroencephalogram
GFAP: Glial fibrillary acidic protein
HHV-6: Human herpesvirus-6
IL: Interleukin
IVIG: Intravenous immune globulin
MERS: Mild encephalitis/encephalopathy with a reversible splenial lesion syndrome
MRI: Magnetic resonance imaging
PCR: Polymerase chain reactions
RESLES: Reversible splenial lesion syndrome
SCC: Splenium of the corpus callosum
